# Surface-Modified Nanocarriers for Nose-to-Brain Delivery: From Bioadhesion to Targeting

**DOI:** 10.3390/pharmaceutics10010034

**Published:** 2018-03-15

**Authors:** Fabio Sonvico, Adryana Clementino, Francesca Buttini, Gaia Colombo, Silvia Pescina, Silvia Stanisçuaski Guterres, Adriana Raffin Pohlmann, Sara Nicoli

**Affiliations:** 1 Interdipartmental Center for Innovation in Health Products, BIOPHARMANET TEC, University of Parma, Parco Area delle Scienze 27/a, 43124 Parma, Italy; adryana.rochaclementino@studenti.unipr.it (A.C.); francesca.buttini@unipr.it (F.B.); sara.nicoli@unipr.it (S.N.); 2 Food and Drug Department, University of Parma, Parco Area delle Scienze 27/a, 43124 Parma, Italy; silvia.pescina@unipr.it; 3 Department of Life Sciences and Biotechnology, University of Ferrara, Via Fossato di Mortara 17/19, 44121 Ferrara, Italy; clmgai@unife.it; 4 Programa de Pós-Graduação em Ciências Farmacêuticas, Universidade Federal do Rio Grande do Sul, Porto Alegre 90610-000, Brazil; silvia.guterres@ufrgs.br (S.S.G.); adriana.pohlmann@ufrgs.br (A.R.P.); 5 Departamento de Química Orgânica, Instituto de Química, Universidade Federal do Rio Grande do Sul, Porto Alegre 91501-970, Brazil

**Keywords:** nose-to-brain delivery, nanoparticles, pharmaceutical nanotechnology, mucoadhesion, mucus-penetrating particles, targeting, CNS disorders, neurodegenerative diseases, Alzheimer’s disease, Parkinson’s disease

## Abstract

In the field of nasal drug delivery, nose-to-brain delivery is among the most fascinating applications, directly targeting the central nervous system, bypassing the blood brain barrier. Its benefits include dose lowering and direct brain distribution of potent drugs, ultimately reducing systemic side effects. Recently, nasal administration of insulin showed promising results in clinical trials for the treatment of Alzheimer’s disease. Nanomedicines could further contribute to making nose-to-brain delivery a reality. While not disregarding the need for devices enabling a formulation deposition in the nose’s upper part, surface modification of nanomedicines appears the key strategy to optimize drug delivery from the nasal cavity to the brain. In this review, nanomedicine delivery based on particle engineering exploiting surface electrostatic charges, mucoadhesive polymers, or chemical moieties targeting the nasal epithelium will be discussed and critically evaluated in relation to nose-to-brain delivery.

## 1. Pharmaceutical Nanotechnologies for Nose-to-Brain Delivery

Among non-conventional routes of drug administration, nasal delivery has undoubtedly received less attention compared to other routes such as the pulmonary and transdermal ones. Traditionally, nasal drug administration has been associated with the treatment of minor local ailments, such as rhinorrhea, nasal congestion, nasal infections, and allergic or chronic rhinosinusitis [1]. However, the advantages of nasal delivery include ease of administration, non-invasiveness, good patient acceptability, rapid onset of action, relatively large and permeable absorption surface, reduced enzymatic activity, and avoidance of hepatic first-pass metabolism. Therefore, the number of products exploiting the nose for systemic delivery of small and large molecules (including peptides, proteins, and vaccines) is increasing in the market. Applications go from smoke cessation (nicotine, Nicotrol^®^ NS, Pfizer, New York City, NY, USA) to flu vaccination (live attenuated influenza vaccine, FluMist^®^ Quadrivalent, Astra Zeneca, Wilmington, DE, USA), from pain management (fentanyl, Intstanyl^®^, Takeda, Japan and Pecfent/Lazanda^®^, Archimedes Pharma Ltd., Reading, UK; butorphanol tartrate spray, Mylan Inc., Canonsburg, PA, USA) to postmenopausal osteoporosis (salmon calcitonin, Fortical^®^, Upsher-Smith, Maple Grove, MN, USA), from the treatment of migraine (zolmitriptan, Zomig^®^, AstraZeneca, Cambridge, UK; sumatriptan, Imigran, GSK, Brentford, UK and Onzetra™ Xsail™, Avanir Pharmaceuticals, Aliso Viejo, CA, USA) to those of endometriosis (nafarelin, Synarel^®^, Pfizer, New York City, NY, USA) or prostate cancer (buserelin, Suprecur^®^, Sanofi-Aventis, Paris, France) [2].

Seemingly, however, the best is yet to come, as the nasal cavity offers a unique opportunity for the delivery of pharmaceutically active ingredients (APIs) to the central nervous system (CNS). Given the increasing incidence of brain diseases and neurological disorders associated with the aging population, achieving efficient drug delivery to the brain is a priority of modern pharmaceutical sciences. However, brain delivery of drugs is challenging, as the CNS is protected by the blood brain barrier (BBB) and the blood cerebrospinal fluid barrier (BCSFB), two structures assuring selective brain permeability to circulating molecules. These physical, metabolic, and transporter-regulated barriers greatly limit the number of APIs able to access the CNS at therapeutic concentrations [3]. Several approaches have been proposed to improve brain delivery across BBB [3,4], including nanoparticulate drug carriers targeting transporters expressed on the BBB [5,6,7]. Unfortunately, the percentage of injected drug dose reaching the brain even with BBB-targeting or permeation-enhancing strategies is below 5%, typically less than 1%, with the remaining 95–99% of the drug off-target and potentially responsible for systemic side effects. Furthermore, in the case of nanocarriers, the CNS chronic toxicity and immunogenicity of polymers, surfactants, and other components must be carefully evaluated, especially considering the generally long treatment duration [8].

**Figure 1:**
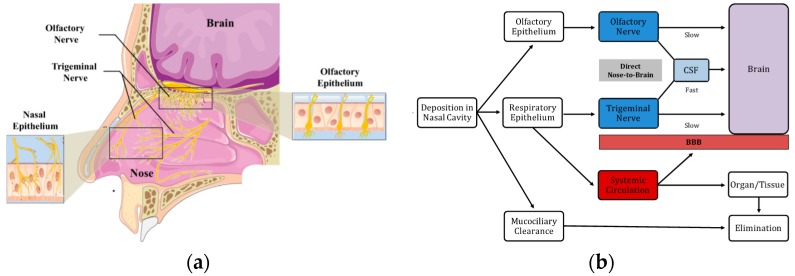
(**a**) Nasal innervation; (**b**) Nose-to-brain (N2B) pathways of drug delivery (modified from [15,16] with permission).

Increasing evidence suggests that intranasal drug delivery enables both small and large molecules to bypass the BBB via the nerves of the nasal cavity, i.e., the olfactory and trigeminal nerves. In particular, the olfactory “neuroepithelium” is the only region of the CNS that is not protected by the BBB and, thus, in contact with the external environment. Consequently, it is a unique access port to the brain [9]. In addition, the trigeminal nerve appears notably involved in the nose-to-brain (N2B) delivery of certain substances, especially towards the posterior region of the brain [10,11]. Thus, following nasal administration, drugs can reach the CNS via three main pathways: (A) The olfactory nerve, which innervates the nasal olfactory epithelium and terminates in the olfactory bulb; (B) The trigeminal nerve, which innervates the respiratory and (to a lower extent) the olfactory epithelium through its ophthalmic and maxillary branches, terminating in the brainstem and olfactory bulb, respectively; and (C) The vascular pathway. Among these, the olfactory and trigeminal nerve pathways provide brain delivery via either a slow intracellular axonal transport (hours or even days) or a fast perineural paracellular transport (minutes) from the sub-mucosal space to the cerebrospinal fluid (CSF) compartment [12,13]. The vascular pathway provides a secondary, indirect mechanism of delivery, whereby the drug is firstly absorbed into the systemic circulation and subsequently enters the brain by crossing the BBB [14]. Figure 1 outlines the nasal innervation and the three brain-targeting pathways of nasal delivery.

Hence, nasal delivery has been proposed for the treatment of various CNS conditions, like migraine [17], sleep disorders [18], viral infections [19], brain tumors [20,21], multiple sclerosis (MS) [22], schizophrenia [23], Parkinson’s disease (PD) [24], Alzheimer’s disease (AD) [25], and even obesity [26]. Potential limitations can arise from the nasal cavity’s small volume (which limits the amount of formulation that can be administered), poor olfactory region deposition from conventional nasal devices, short residence time, low bioavailability of hydrophilic and/or large molecules, mucosal irritation, and lack of validated translational animal models [27]. All these may negatively affect nose-to-brain transport, to the point that some authors in the mid 2000s questioned that N2B could be exploited successfully in humans [28,29].

Since then, nasal devices enabling the deposition of a nasal formulation in the nasal olfactory region have been designed: ViaNase atomizer (Kurve Technologies, Mill Creek, WA, USA), pressurized Precision Olfactory Device (Impel Neuropharma, Seattle, WA, USA), and the liquid and powder Exhalation Delivery Systems (OptiNose, Yardley, PA, USA) are currently available for the development of new medicinal products [30].

Preclinical studies in animals increasingly use specific indexes to quantify the efficiency of brain delivery following nasal administration, such as the nose-to-brain drug targeting efficiency (DTE, Equation (1)) and the direct transport percentage (DTP, Equation (2)) [31]. The first index expresses the exposure of the brain to the drug after nasal administration relative to that obtained by systemic (intravenous) administration:

(1) DTE= (AUCBrainAUCBlood)IN(AUCBrainAUCBlood)IV·100

where *AUC_Brain_* and *AUC_Blood_* are the area under the concentration versus the time curves of the drug in the brain and in the circulation (blood, plasma, or serum), respectively, after intranasal (IN) and intravenous (IV) administration. DTE values range from 0 to +∞: values above 100% indicate a more efficient brain targeting after IN than after IV administration.

The direct transport percentage index estimates the fraction of the IN dose reaching the brain via direct nose-to-brain transport versus the total amount of drug reaching the brain after intranasal delivery:

(2) $$\mathrm{DTP}=\;\frac{B_{IN}-\;B_{x}}{B_{IN}}\cdot 100$$

where *B_IN_* is the brain AUC following intranasal administration, and *B_x_* is the fraction of the same AUC accounting for the drug that crossed the BBB from the systemic circulation. *B_x_* can be calculated according to Equation (3):

(3) $$B_{x}=\;\frac{B_{IV}}{P_{IV}}\cdot P_{IN}$$

where *P_IN_* and *P_IV_* are the blood AUC after intranasal and intravenous administration, respectively. Positive DTP values up to 100% indicate a contribution of the direct nose-to-brain pathways to brain drug levels, whereas DTP equal to 0 (or even negative) indicates that the drug preferentially enters the brain via the systemic circulation after IV administration. These quantitative preclinical pharmacokinetics (PK) data, associated with pharmacodynamics (PD) data, allow to build advanced translational PK and PK-PD models to predict CNS concentrations in humans [32].

In parallel, some clinical trials on nasal drug delivery for brain targeting have been carried out in humans, including insulin for Alzheimer’s disease [33,34], oxytocin for autism [35], schizophrenia, and major depressive disorder [36], and davunetide for mild cognitive impairment [37,38] and progressive supranuclear palsy [39]. These trials prove that N2B delivery is indeed taken into consideration by pharmaceutical companies as a promising clinical approach [40].

Despite the advancements in the field, the delivery of drugs presenting unfavorable physicochemical and biopharmaceutical characteristics, such as rapid chemical or enzymatic degradation, poor aqueous solubility, low permeability, and low potency, requires a formulation able to enhance drug transport to the brain, without disrupting the structure and physiology of the nasal epithelium.

Pharmaceutical nanotechnologies can be strategic for the formulation and N2B delivery of these substances, including peptide and proteins. In fact, nanosized (1–1000 nm) drug delivery systems can:

- Protect the encapsulated drug from biological and/or chemical degradation
- Increase the drug apparent aqueous solubility
- Enhance the residence time at the site of absorption
- Promote mucosal permeation and/or cellular internalization
- Control the release kinetics of the encapsulated drug
- Achieve targeted drug delivery through surface modification with specific ligands
- Reduce the drug distribution to non-target sites, minimizing its systemic side effects.

All these features are desirable for efficient N2B delivery and represent critical issues toward the effective application of drugs that per se (i.e., without a carrier) would not achieve CNS concentrations leading to a pharmacological effect. Therefore, almost any pharmaceutical nanocarrier has been studied for nose-to-brain delivery, including nanocrystals [41,42], micelles [43,44], liposomes [45], solid lipid nanoparticles (SLN) [46,47], nanostructured lipid carriers (NLC) [48,49], polymeric nanoparticles [50,51,52], albumin nanoparticles [53], gelatin nanoparticles [53], dendrimers [54], mesoporous silica nanoparticles [55], nanoemulsions [56].

The research activity in this field has been extensively reviewed in recent publications on the use of nanocarriers for N2B delivery, covering both general [57,58,59,60,61] and specific disease [62,63] or carrier-related topics [64,65,66]. The present review does not aim to provide an exhaustive report on the nasal use of nanoystems for direct drug delivery to the brain. Conversely, it reviews and critically appraises some facts and figures about the leading strategies of nanoparticle (NP) design for nose-to-brain delivery. In particular, this review will focus on nanoparticle physicochemical characteristics and their surface modification with mucoadhesive, penetration-enhancing or targeting moieties, able to influence and promote drug brain delivery.

## 2. Influence of Physicochemical Properties on Nanoparticles Nose-to-Brain Delivery

Many papers describe enhanced brain delivery after nasal administration of nanoencapsulated drugs in comparison with free drug formulations. However, few studies investigate the mechanism by which nanoparticles enhance drug transport to the brain. Different scenarios can be depicted; the simplest one implies that the nanocarriers interact with the mucus layer and release the drug in the mucus or at the mucus–epithelial cell interface. The most “challenging” scenario implies that the drug-loaded nanoparticles themselves cross the mucosal barrier, are taken up by neurons and translocated along the nerve axons (trigeminal and olfactory) to reach the brain, where the drug is released. The option in-between the two described involves nanoparticle uptake into the nasal respiratory epithelium and/or through the olfactory neuroepithelium, where the drug payload is released. Then, the free drug diffuses along perineural spaces to the CNS. It is clear that the nanoparticle fate depends on their own physicochemical characteristics. In general, composition, shape, size, surface charge, and surface hydrophobicity/hydrophilicity affect the nanocarrier interaction with the biological environment. In N2B delivery, these features influence the interaction with mucus, the uptake by the epithelial and neuroepithelial cells, the translocation to the brain by diffusion along the axons, and the release kinetics of the drug. In this context, the elucidation of the role of the physicochemical properties of nanoparticles is essential to design efficient and safe drug carriers.

In order to shed light on the role of NP characteristics such as particle size, surface charge, hydrophobicity on their fate, some authors have studied transport either in vitro across olfactory cell monolayers, ex vivo across excised nasal mucosa, or in vivo with rodent models.

In a recent paper, Gartziandia et al. [67] compared the permeability of nanoparticles having different physicochemical properties across primary cell monolayers of rat olfactory mucosa. A fluorescent probe (DiR; 1-1′dioctadecyl-3,3,3′,3′-tetranethylindotricarbocyanine) was loaded into the nanoparticles to track them, after demonstrating no probe release in the transport buffer. Significant differences in nanoparticle permeation were observed as a function of the material the particles were made of: nanostructured lipid carriers (NLCs) penetrated to a higher extent compared to poly(lactic-*co*-glycolic acid) (PLGA) nanoparticles having the same zeta potential (−23 mV). The change of the surface potential of NLCs from negative to positive by chitosan coating increased the transcellular transport by almost threefold compared to the uncoated NLCs. Finally, surface functionalization using cell-penetrating peptides (in particular Tat) further enhanced nanoparticle transport. While the role of chitosan can be explained considering an electrostatic interaction with the negatively charged cells, the different performance observed deserves further investigation before attributing it to the nanoparticle composition, i.e. polymer versus lipid. Indeed, the studied particles had different sizes (approx. 100 nm for NLCs and 220 nm for PLGA nanoparticles) and relied on different surfactants for their preparation, namely, polysorbate 80 and poloxamer for NLCs (PEG moieties were found on NP surface) and poly(vinyl alcohol) (PVA) for PLGA nanoparticles. This may have contributed to the different penetration observed, considering the mucus-penetrating effect of PEG (see also Section 4.1) and the mucoadhesive properties of PVA-coated particles. The latter have been reported to interact with mucus components by hydrogen bonding and/or hydrophobic interactions [68].

Musumeci and collaborators [69] prepared PLGA, PLA, and chitosan nanoparticles using polysorbate 80 (Tween 80) as a surfactant and rhodamine as a fluorescent probe. They found a higher uptake in olfactory ensheathing cells (extracted from rat pups’ olfactory bulbs) for PLGA NP (132 nm, −15.8 mV) compared to chitosan (no surfactant, 181 nm, +34 mV) and PLA (152 nm, −30 mV) nanoparticles. The authors explained the higher uptake of PLGA nanoparticles considering the lower absolute surface charge, but the presence of PEG moieties on PLGA and PLA particle surface could have contributed as well to this result. However, it is difficult to compare the data from the two previously cited studies because different cells were used. It is known that the type and the physiological status of a cell highly influence its behavior with respect to nanoparticle uptake [70].

Mistry et al. [71] chose a more complex barrier, i.e., excised porcine olfactory epithelium mounted on Franz-type diffusion cells, to compare the behavior of carboxylate-modified fluorescent polystyrene nanoparticles measuring 20, 100, and 200 nm in size (ζ potential: approx. −42 mV) with that of surface-modified nanoparticles obtained by coating with chitosan (48, 163, or 276 nm; ζ potential approx. +30 mV) or polysorbate 80 (ζ potential approx. −21 mV). None of the tested particles crossed the nasal epithelium after 90 min, but polysorbate 80-coated (PEGylated) particles penetrated deeper in the tissue compared to uncoated and chitosan-coated nanoparticles. On the other hand, the number of particles present at the epithelial surface was higher in the case of chitosan-coated particles, and histological images suggested a localization within the mucus layer. No clear trend was found concerning the influence of nanoparticle size on drug uptake into the tissue.

The same nanoparticles were also evaluated in vivo in a mouse model [72]. An amount of 15 µL of a formulation containing one type of NP, i.e., 105 nm polystyrene nanoparticles (−42 V), 163 and 276 nm chitosan-coated NP (ζ potential +30 and +23 mV, respectively), or 107 and 180 nm polysorbate 80-coated NPs (ζ potential −21 and −24 mV, respectively) was applied daily for 3 days. All nanoparticles were transported to some extent across the mucosa (both olfactory and respiratory) via a transcellular route. The presence of polysorbate 80 coating did not enhance tissue uptake as compared to the uncoated particles, despite the claimed mucus-penetrating properties of PEG. The authors explained these results considering that the PEG chains were not covalently bound to the particles, and that a precise length and density of PEG chains are needed to obtain a relevant mucus-penetrating effect [73,74]. A significant difference was found between 107 and 180 nm polysorbate 80-coated NPs, and the lower accumulated amount measured for the larger particles was attributed to their slower diffusion across the mucus network. Chitosan-coated particles were mainly retained inside the mucus, and lower amounts were found in the tissue in comparison with uncoated and polysorbate 80-coated nanoparticles. Despite the long application time (4 days) the nanoparticles were never found in the olfactory bulb, regardless of their size and superficial properties.

Ahmad [75] studied the permanence of a nanoemulsion made with Labrafac^®^WL1349/Labrafac^®^CC (Gattefossé, Saint-Priest, France) and Solutol^®^HS15 (BASF, Mumbai, India) in the nasal cavity of rats from 0.5 to 16 h after the application of 100 µL of formulation. In particular, nanoemulsions with droplets of 80, 200, 500, and 900 nm (NE80, NE200, NE500 and NE900) were compared. The droplets were tracked with environment-responsive probes giving a fluorescent signal when dispersed in the nanocarrier matrix that is then quenched immediately after release. It was found that the smaller the droplet size, the higher the retention time in the nasal cavity. The even longer retention obtained with positively charged chitosan-coated nanodroplets (size 108 nm) was attributed to the electrostatic interaction with the mucus and the negatively charged epithelial membranes. Further studies were carried out with NE80, NE900 uncoated nanoemulsions, and the 108 nm chitosan-coated nanoemulsion. After 1 h from nasal instillation, a large number of nanoemulsion droplets were present in the nasal mucosa, with the highest signal given by the chitosan-coated nanoemulsion, followed by NE80 and NE900 uncoated nanoemulsions. In the trigeminal nerve, the translocation was size-dependent, with NE80 > Chitosan coated > NE900. The accumulation in the brain (and in particular in the olfactory bulb) was also assessed: very few nanoemulsion droplets entered the brain after 1 h and were only visualized in the case of small-size particles (NE80 and 108 nm, chitosan-coated), in agreement with the in vivo results obtained by Mistry [72].

In contrast, a recent paper reported significant brain accumulation of PLGA nanoparticles after nasal administration in rats. The authors prepared rhodamine-loaded PLGA nanoparticles (surfactant: polysorbate 80, size 118 nm, ζ potential −26 mV) and chitosan-PLGA nanoparticles (213 nm, +69 mV) and analyzed the brain distribution of fluorescence after 8, 24, or 48 h from intranasal administration. The results evidenced that both positively and negatively charged particles reached the brain and were localized mainly in the cytosol of neural cells. A different localization (caudal versus rostral area) could be obtained by modifying the surface charge. A particle distribution depending on the post-application time was also described, with slower brain uptake for positively charged particles, which was attributed to both mucus–nanoparticle interactions in the nasal cavity and different nose-to-brain pathways. The authors hypothesized that the slower translocation of the positive particles exploited an intra-neuronal pathway (trigeminal nerve), whereas the extra-neuronal pathway, relying on bulk flow transport, was responsible for the rapid transport of the negatively charged particles. Despite the interesting results, no experimental evidence, with the exception of the different timing of brain appearance, supported this hypothesis, and the involvement of different (systemic) pathways could not be excluded.

**Figure 2:**
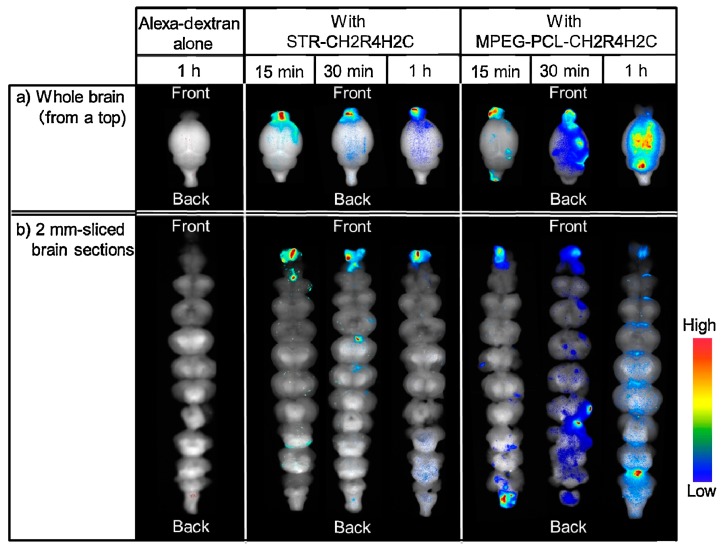
Dynamics of Alexa-dextran in brain tissue following nasal administration of hydrophobic (STR–CH2R4H2C) and hydrophilic (MPEG–PCL–CH2R4H2C) surface nanocarriers in: (**a**) whole brain and (**b**) 2 mm-sliced brain sections (reprinted with permission from [76]).

A relationship between brain distribution and nanocarrier properties was also described by Kanazawa et al. [76] by using peptide-based carriers. An arginine-rich oligopeptide (designed to have adhesiveness and transmissibility) was conjugated with either a hydrophobic moiety (stearic acid) or a hydrophilic one (PEG–PCL block copolymer) in order to obtain two stable micellar formulations. An Alexa-dextran complex (*M*_W_ 10,000 Da) was used as a fluorescent probe to assess the biodistribution. The stearate–peptide and PEG–PCL–peptide micelles measured 100 and 50 nm in size and had a ζ potential of +20 and +15 mV, respectively. After intranasal application in rats, the two carriers determined a much higher uptake in the nasal mucosa and the brain as compared to Alexa-dextran alone. The hydrophobic stearate–peptide determined a significantly higher fluorescence in the nasal epithelium as compared to the hydrophilic PEG–PCL–peptide, but a lower fluorescence in the trigeminal nerve. Additionally, when analyzing the intracerebral distribution pattern of Alexa (Figure 2) as a function of the time post-intranasal administration, a significant difference between the two carriers could be appreciated: the hydrophobic stearate–peptide showed a strong fluorescence in the forebrain after 15 min, 30 min, and 1 h, and no transport to the hindbrain was evidenced. In contrast, upon PEG–PCL–peptide administration, a spreading of the fluorescence after 30 min and 1 h was evident, indicating a distribution of Alexa in the entire brain. This result, together with the higher trigeminal fluorescence, suggests that PEG–PCL–peptide nanocarriers penetrated across the nasal mucosa and transported the probe to both the olfactory bulb (forebrain) and to the hindbrain via the olfactory and trigeminal nerves.

Gabal et al. [77] evaluated the impact on N2B delivery of the nanoparticle surface charge by preparing anionic and cationic NLCs having very similar sizes (175 nm and 160 nm, respectively) and opposite ζ potential values (−34 and +34 mV, respectively). The particles contained the same amount of drug (ropinirole HCl, encapsulation efficiency EE% 53 and 50%, respectively) and showed very similar release kinetics in vitro. These nanoparticles were dispersed in a thermosensitive gel made of poloxamers (407 and 188) and hydroxypropyl methylcellulose (HPMC) and administered intranasally to albino rats. The animals were sacrificed 3 to 360 min after the intranasal application, and the drug levels were measured in the plasma and brain to calculate the pharmacokinetic parameters. Overall, there was no significant difference between negative and positive nanoparticles, and both performed much better than a drug solution. However, the actual contribution of the nanocarrier is not clear since the improved bioavailability appears mainly due to the increased residence time resulting from the presence of the gel and/or of a penetration enhancer (sodium deoxycolate) in the nanoparticle formulation. Indeed, the authors also compared the toxicity of anionic NLCs, cationic NLCs, and the same nanocarriers dispersed in the gel after daily 10 µL applications for 14 days in rats. The results highlighted the highest toxicity for cationic NLC, followed by anionic NLCs, whereas no histopathological alterations were found in animals treated with gels loaded either with cationic or with anionic particles. The authors attributed this finding to a direct protective mechanism of poloxamer 188 against oxidative stress and inflammation. However, the hindered nanoparticle diffusion through the gel network and the consequent limited interaction between the nanoparticles and the epithelium may be considered as the real reasons of the observed reduced toxicity.

The potential toxicity of nanoparticles to the nasal mucosa and also to CNS structures is a critical point to consider in nose-to-brain applications. In agreement with the abovementioned results, also other authors described a higher toxicity of positively charged nanoparticles: chitosan-coated nanoparticles applied in a pH 6.0 citrate buffer vehicle had a size-dependent damaging effect on excised porcine olfactory epithelium: 20 nm nanoparticles caused substantial tissue damage as compared to 100 and 200 nm nanoparticles [71]. This effect can be partially attributed to the buffer but also to the high relative surface area, combined with the presence of the positive surface charge. However, it is worth mentioning that toxicity studies on animal models have evidenced a substantial safety of nanoparticles made of or coated with chitosan [78]. This suggests that in vivo the presence of a thicker mucus layer can reduce the nanoparticle toxic effects, probably by reducing the interaction between the nanoparticles and the epithelium.

Extensive nanotoxicological literature showed the capability of pollutants and metal nanoparticles to reach the brain parenchyma after nasal instillation or inhalation and, in some cases, to elicit toxic effects in the CNS [79,80,81]. Thus, a careful selection of the excipients used is mandatory, and a rapid and efficient biodegradation within the absorption tissue appears the best strategy to avoid unwanted accumulation and potential CNS toxicity of the innovative nanocarriers.

## 3. Mucoadhesive Nanoparticles

Mucociliary clearance is a major physiological factor that significantly impacts on nasal drug delivery and N2B transport. This protection mechanism of the respiratory apparatus efficiently and rapidly eliminates noxious substances, particulate matter, and microorganisms trapped in the mucus layer (10–15 µm thick) after intake by air (clearance *t*_1/2_ 20 min in humans). However, this system greatly limits the residence time of substances administered inside the nasal cavity. As a consequence, traditional nasal formulations exploit excipients able to increase viscosity and/or provide bioadhesion, such as hydrophilic polymers, to counteract mucociliary clearance, prolong formulation residence time, improve systemic bioavailability, and reduce nasal absorption variability [82]. The mucus lining the nasal epithelium is secreted by goblet cells of the epithelium and mainly by submucosal glands present in the lamina propria. It is composed of about 90–95% water, 2–5% mucins (proteins), 1% salts, and variable amounts of cellular products and debris, such as DNA, albumin, immunoglobulins, lysozyme, lactoferrin, and lipids [73,83]. In particular, the highly glycosylated mucins (10–40 MDa) are the main responsible of mucus properties. Owing to their molecular weight, hydration, and entanglement, these glycoproteins give the nasal secretion its typical viscosity and elasticity (non-Newtonian thixotropic gel). In addition, mucins contain high levels of sialic acid and sulfate residues, which provide a neat negative charge to the polymer chains that contributes to the rigidity of their networks [73]. In the case of polymeric excipients, mucoadhesion has been described as a plethora of events, together leading to the actual adhesion: hydration of polymer chains, intimate contact with the mucus, diffusion and entanglement with mucin fibers, dynamic creation and disruption of labile bonds such as disulfide bridges, electrostatic attractive forces, hydrophobic interactions, hydrogen and van der Waals bonds [84]. Several natural (gums, alginates, starch, and gelatin), semisynthetic (cellulose derivatives, such as methyl−, hydroxylpropyl–, hydroxypropylmethyl–, and carboxymethylcellulose), and fully synthetic polymers (polyacrylates, polymethacrylates, crospovidone) have been used to improve the nasal delivery of drugs [83]. Concerning N2B transport, for example, chitosan and low-molecular-weight pectins were shown to prolong the residence time of nasal formulations in the olfactory region in humans [85]. Sodium hyaluronate improved the brain delivery of a high-molecular-weight hydrophilic model compound (fluorescein-labeled 4 kDa dextran) after nasal administration to rats [86].

**Figure 3:**
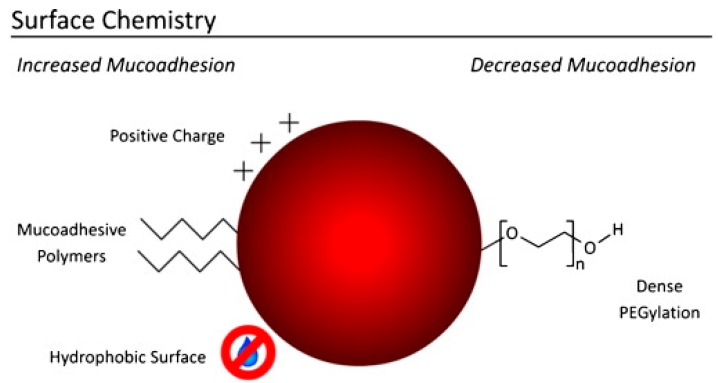
Surface chemistry of nanoparticles having an influence on mucoadhesion (modified with permission from [84]).

When these polymers are main constituents or surface-modifiers of nanocarriers, the underlying adhesion mechanisms do not change, but the carrier’s high surface-area-to-volume ratio translates into an extensive interface for more stable and prolonged interactions with the mucus compared to larger structures. Besides, a size below 500 nm allows the nanoparticles to squeeze in the non-viscous aqueous pores within the entangled mucin network, further enhancing the interaction with the mucus at a molecular level. Finally, positive and hydrophobic surfaces may contribute to maximize nanoparticle adhesion to the mucus, based on electrostatic attraction and hydrophobic interactions with mucins’ negatively charged and hydrophobic domains, respectively [84]. However, some surface modifications reduce mucoadhesion, and this characteristic is exploited by mucus-penetrating nanocarriers, as discussed in next section of this review (Figure 3).

For the above reasons, mucoadhesive nanocarriers have been extensively studied for N2B delivery of drugs.

In the year 2000, Betbeder and coworkers proposed Biovector™ nanoparticles for nasal delivery of morphine exploiting the direct pathway between the olfactory mucosa and the CNS. The nanocarriers were maltodextrin-based cationic nanoparticles surrounded by a phospholipid bilayer (average size 60 ± 15 nm). They prolonged morphine antinociceptive activity when co-administered with the opioid in mice in comparison to a morphine solution. Interestingly, the result was not replicated when morphine was administered with a penetration enhancer such as sodium deoxycholate. Despite morphine was actually not nanoencapsulated, indeed nanoparticles improved its nose-to-brain transport, while blood levels were not significantly affected. This was likely due to the interaction of the positive nanoparticles with the nasal mucus layer, which may have increased the formulation residence time and absorption from the olfactory region that is particulary developed in mice [87].

Another approach was proposed by the group of Silvia Guterres and Adriana Pohlmann, who developed amphiphilic methacrylic copolymer-functionalized poly(ε-caprolactone) nanocapsules as a mucoadhesive system for N2B delivery of olanzapine, an atypical antipsychotic drug [88]. Nanocapsules showed pH-dependent size and surface charge values, ranging from 324.3 to 235.2 nm and from +55 and +22.7 mV. The nanocapsule–mucin interaction was evidenced on the basis of the increase in particle size and reduction in nanoparticle concentration observed using Nanoparticle Tracking Analysis (NTA). The polymer mucoadhesion was tested in terms of peak force, work of adhesion, and prolongation of tehe residence time of olanzapine-loaded nanocapsules on porcine nasal mucosa. In vivo studies in rats confirmed a 1.55-fold higher accumulation of olanzapine in the brain after nasal administration of mucoadhesive nanoparticles in comparison to a drug solution used as a control. Furthermore, the amphiphilic methacrylic copolymer-functionalized poly(ε-caprolactone) nanocapsules loaded with olanzapine outperformed the controls (drug solution and blank nanocapsules) also in the pre-pulse inhibition model of cognitive impairment symptoms typical of schizophrenia. Interestingly, the authors noted that brain accumulation was higher than that reported in the literature for olanzapine-loaded PLGA nanoparticles and attributed such improvement to the cationic mucoadhesive coating of the nanocapsules [88].

Notwithstanding the possibilities offered by new synthetic polymers, polysaccharides have been amongst the most popular materials used to build mucoadhesive nanocarriers. In fact, polysaccharides are ideal for the production of nanoparticles for nasal delivery not only because of their mucoadhesive properties, but also because of their unique characteristics, such as biomimetic mucosal recognition, biocompatibility and biodegradability, and ease of chemical modification with targeting moieties. In this regard, polysaccharides can be incorporated into pharmaceutical nanocarriers in three ways: by absorption to preformed nanoparticles, by copolymerization or covalent grafting leading to surface modification, or by directly manufacturing polysaccharide-based nanoparticles [89]. For example, albumin nanoparticles obtained by coacervation and thermal cross-linking were prepared in the presence of β-cyclodextrin derivatives in order to develop an innovative nasal drug delivery system for the anti-Alzheimer drug tacrine. The inclusion of β-cyclodextrin derivatives affected drug loading and modulated nanoparticle mucoadhesiveness. Finally, tacrine permeation across sheep nasal mucosa from tacrine-loaded nanoparticles modified with β-cyclodextrin derivatives increased compared with unmodified albumin nanoparticles, but was lower than that obtained with a drug solution. This can be explained considering the prolonged release of tacrine observed in the case of nanoparticles [90].

Surface-engineered nanostructured lipid carriers coated with *Delonix regia* gum (DRG–NLC) as a natural mucoadhesive polymer were proposed for N2B delivery of ondansetron (OND), a centrally active drug used for the management of chemotherapy-induced nausea and vomiting. The OND mucoadhesive formulation was manufactured by high-pressure homogenization using glycerol monostearate and Capryol^®^ 90 as solid and liquid lipids, while soybean lecithin and poloxamer 188 were used as stabilizers. The nanostructured lipid carriers were subsequently coated by dispersing OND-loaded nanoparticles in a 0.75% (*w*/*v*) DRG aqueous solution. The optimal nanoparticle formulation showed an average size of 92 nm, negative surface charge, and acceptable encapsulation efficiency. Mucoadhesion was assessed in vitro by determining the binding efficiency (%) of DRG-NLC toward mucin (72%). In vivo studies in rats showed a brain targeting efficiency (DTE) of 506% and a direct transport percentage (DTP) of 97% for the intranasal administration of OND-loaded DRG–NLC versus the IV administration of a commercial OND injection (Emeset^®^) as a control. Despite the remarkable results, this study lacked a nasal control (non-mucoadhesive formulation) and did not clarify the impact of the presence of free gum and of non-encapsulated drug, as well as of the processing of nanoparticles (freeze–drying) on the in vivo brain distribution obtained after DRG–NLC nasal delivery [91].

Alginate nanoparticles were produced for N2B delivery of venlafaxine, a serotonin and norepinephrine reuptake inhibitor, used for depression. Alginate nanoparticles were prepared by ionotropic gelation with calcium ions and subsequent cross-linking with a polycation, namely, low-molecular-weight chitosan glutamate. Optimized nanoparticles showed an average size slightly below 175 nm and positive surface charge, with a high encapsulation efficiency and a drug loading of nearly 27%. Mucoadhesion was not studied directly, but ex vivo permeation across porcine nasal mucosa over 24 h was twice as higher with venlafaxine-loaded nanoparticles compared to a drug solution. The forced swimming test and the locomotor activity test were used as behavioral tests to assess the efficacy of the nasal administration of the antidepressant nanoformulation using two controls: (1) a drug solution administered intranasally and (2) a drug suspension obtained by crushing commercial tablets, administered orally. The alginate nanoparticle formulation performed better than both controls in depressed animals, even if the climbing and immobility parameters were not restored to the levels of naive animals. It must be noted also that, because of the relatively high volume of nanoparticle suspension administered intranasally (100 µL), the possible inhalation or swallowing of part of this nanoformulation could not be excluded. This would eventually provide confounding effects to the final results. In any case, pharmacokinetics studies conducted using venlafaxine IV injection as a reference and venlafaxine IN solution as a control, showed reduced blood levels and increased brain concentration for the antidepressant drug formulated in the alginate nanoparticles. In particular, the DTE and DTP calculated for the venlafaxine-loaded nanocarrier were 426% and 76%, respectively. An increase in absorption as a consequence of reduced nasal mucociliary clearance, an enhanced mucosal permeation, and a modulation of P-gp efflux transporters are possible mechanisms explaining the pharmacokinetics data [92]. It is remarkable, however, that the nanoparticles increased the DTP value only from 63% to 76%, when compared to the nasal solution and that a significant contribution to DTE increase came from the BBB crossing of the drug after systemic absorption. This is not unexpected, as this antidepressant drug is already marketed as immediate and controlled release tablets and crosses the BBB. Thus, the real improvement offered by N2B delivery would mainly consist in the reduction of systemic side effects.

Among polysaccharides, chitosan has been demonstrated a very promising and versatile materials for N2B delivery. It is the copolymer of glucosamine and *N*-acetyl glucosamine and is obtained by deacetylation of chitin. Chitosan not only is biocompatible, biodegradable, mucoadhesive, and positively charged at nasal slightly acidic pH, but is also an efficient permeation enhancer able to transiently open the tight junctions between epithelial cells in mucosal tissues [78,93]. Interestingly, it was demonstrated that this property is retained when chitosan is used as the main component or surface coating agent for nanocarriers [84,94].

Wang and co-workers loaded estradiol in chitosan nanoparticles produced by ionotropic gelation for nasal administration in Alzheimer’s disease. Chitosan nanoparticles were obtained using tripolyphosphate anions (TPP) and chitosan with *M*w 50,000 Da, with particle size of about 270 nm and positive surface charge (+25 mV). The encapsulation efficiency was around 60% (estradiol concentration 1.9 mg/mL). Smaller (below 100 nm) or larger (500 nm) nanoparticles could be obtained with 6000 Da and 200,000 Da chitosan, respectively, but were considered unsuitable for the intended application. In vivo studies in rats showed a DTE of 320% and DTP of 68% on the basis of the measurement of estradiol concentration in the cerebrospinal fluid (CSF) after IN or IV administration of the estradiol-loaded chitosan nanoparticles. The results were explained by the ability of chitosan nanoparticles to bind mucins and enhance paracellular transport [95]. However, in these experiments, the nasal cavity was isolated from the respiratory and gastrointestinal tracts, a surgical practice reducing the likelihood of interfering absorption from other organs, but also altering the retention time and hence the absorption from the nasal mucosa. In addition, paracellular transport is not expected to affect significantly the mucosal permeation of highly lipophilic compounds like estradiol.

Similar chitosan nanoparticles obtained by ionotropic gelation with TPP have been proposed by several authors for the for N2B delivery of very different drugs, i.e., rivastigmine [96] and thymoquinone [97] for Alzheimer’s disease, bromocriptine [98], ropinirole [99], rasagiline [100]. and pramipexole [101] for Parkinson’s disease, and tapentadol for chronic pain management [102]. Despite the different physicochemical characteristics of the studied drugs, the results shared striking similarities.

Chitosan presents desirable features but also some limitations; for example, it is insoluble at physiological pH and is positively charged only in acidic conditions. These features may potentially interfere with bioadhesion. Hence, several authors chose to work with nanocarriers based on chitosan derivatives. Trimethylchitosan (TMC), for example, is a water-soluble, permanently positively charged chitosan derivative and was used to encapsulate the analgesic neurotransmitter leucine-enkefaline (Leu-Enk) by ionotropic gelation. TMC nanoparticles were able to increase 35 times the permeability of the peptide across porcine nasal mucosa and produced a significant increase of the antinociceptive effect in mice after nasal administration (hot plate and acetic acid-induced writhing tests) [103]. Thiolated chitosan can increase mucoadhesion via the formation of covalent bonds, namely, disulphide bridges, between its thiol groups and mucus glycoproteins [104]. Cyclobenzaprine (CB) and tizanidine (TZ), two centrally acting muscle relaxant drugs used for pain management, were loaded into thiolated chitosan nanoparticles obtained by gelation with sodium alginate. The thiolated nanoparticles showed enhanced permeation and reduced toxicity in the RPMI2650 cell model of human nasal epithelium, increased brain uptake, and significantly enhanced the antinociceptive activity of both drugs when compared to non-thiolated nanoparticles administered nasally [105,106]. In another study, in which the antidepressant selegiline HCl was encapsulated in thiolated chitosan nanoparticles (215 nm, EE% 70), the authors claimed improved anti-inflammatory and neuroprotective effects for the thiolated polysaccharide itself. In in vivo experiments in depression-induced rats, thiolated nanoparticles were superior to control chitosan nanoparticles when administered at the same dose (10 mg/kg). However, the differences in the pharmacodynamics effects were not significant compared to controls when the dose administered was halved (5 mg/kg) [107]. In another research, Di Gioia and co-workers assessed the ability of nanoparticles made of another chitosan derivative to deliver dopamine to the striatum. Glycol chitosan, a water-soluble derivative of chitosan, was used to manufacture nanoparticles by ionotropic gelation with TPP along with sulfobutylether-β-cyclodextrin to improve dopamine loading. When administered intranasally in a single dose, the ananoparticles did not modify the neurotransmitter’s brain levels, whereas repeated intranasal administrations significantly increased dopamine levels in the ipsilateral striatum [108].

It is worth mentioning that in almost all these studies, the nanocarriers improved the performance of drugs that already showed favourable N2B transport when administered nasally in the form of conventional liquid formulations such as solutions.

Other formulation approaches use chitosan as a surface modifier of nanocarriers made of other materials. The water-soluble antipsychotic chlorpromazine HCl was loaded into chitosan-grafted PLGA nanoparticles for the nasal treatment of schizophrenia, aiming to provide brain targeting and sustained drug release, decrease the dose and administration frequency, and reduce the side effects. PLGA nanoparticles were prepared with a combined self-assembly–nanoprecipitation method in the presence of dextran sulphate, followed by the grafting of chitosan on the PLGA nanoparticle surface. The selected formulation, characterized by an average size of 464 nm and 37% encapsulation efficiency, showed good mucoadhesion on sheep nasal mucosa and led to about 9% chlorpromazine permeation over 4 h, controlled by the release kinetics of the PLGA nanoparticles (40% release in 48 h) [109]. Liposomes coated with a chitosan derivative were proposed for the nasal administration of ghrelin. Ghrelin is a centrally acting peptide hormone able to stimulate food intake. For this reason, it is a potential drug candidate for the treatment of cachexia, i.e., the wasting pathologic syndrome associated with some chronic diseases, such as cancer, heart, or renal failure. Ghrelin-loaded liposomes were prepared by the lipid film re-hydration–extrusion technique followed by coating with *N*-([2-hydroxy-3-trimethylammonium]propyl) chitosan chloride. The resulting chitosan-coated liposomes bound mucin more efficiently than the uncoated anionic liposomes (63% versus 40%) and improved permeation through a Calu-3 cell monolayer used as model of the upper airways epithelial barrier (10.8% versus 3.6% uncoated liposomes versus 0% free peptide) [110].

**Figure 4:**
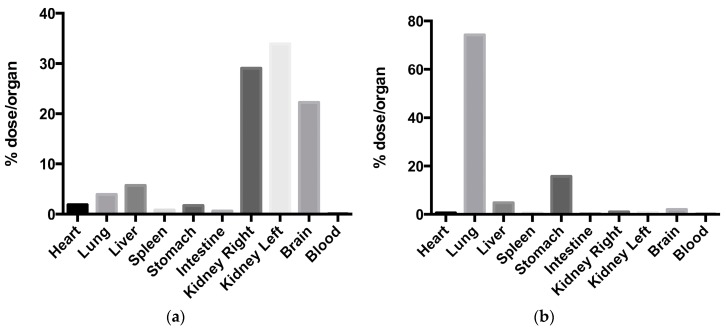
Fraction of total radioactivity recovered per organ 90 min after nasal administration to rats of (**a**) ^99m^Tc-labelled simvastatin-loaded nanoparticles; (**b**) ^99m^Tc-labelled simvastatin suspension [113].

Finally, Clementino and co-workers developed hybrid chitosan–lipid nanocapsules for N2B delivery of simvastatin. Statins have been suggested as potential neuroprotective drugs by reason of their pleiotropic effects, i.e., anti-inflammatory, antioxidant, and immunomodulatory [111,112]. The nanocapsules were obtained by self-assembly of a mixture of phospholipids and liquid lipids added to a chitosan aqueous solution. The nanocapsules obtained not only had small particle size (200 nm), positive surface charge, and high encapsulation efficiency, but also were shown to be efficiently biodegraded by enzymes present in nasal secretions, such as lysozyme. The biodegradation can provide a more efficient release of the encapsulated drug after deposition on the nasal mucosa. ^99m^Tc-labelled simvastatin-loaded nanocapsules were administered intranasally to rats and compared with ^99m^Tc-labelled statin in suspension. Gamma scintigraphy studies evidenced a significantly higher brain accumulation of the isotope (above 20% of the administered radioactivity) administered with the nanocapsules (Figure 4) [113].

Recently, several researchers have proposed the inclusion of various nanoformulations (micro/nanoemulsions, lipid, or polymer nanoparticles) within mucoadhesive gels (xantan gum, chitosan) [114,115] or in situ gelling systems obtained with thermosensitive (poloxamers 407) [116], pH-sensitive (Carbopol 934) [117] and ion-sensitive (gellan gum) [118,119] polymers. Even if the approach is seemingly appealing (combination of the nanoparticle encapsulation/protection with a viscous vehicle to prolong the nasal residence time), the gel matrix viscosity might hinder nanoparticle migration, lead to poor interaction with the nasal epithelium, and down drug release. In addition, the superiority of this approach over more conventional formulations is open to question.

In summary, mucoadhesive nanoparticles have been among the most explored platforms for the N2B delivery of drugs. The available data are generally evidencing a superiority of the mucoadhesive nanoformulations over drug liquid formulations administered nasally and/or intravenously, despite only few studies directly compared mucoadhesive nanocarriers with potentially competing formulations such as liquid or even solid dosage forms containing mucoadhesive or permeation-enhancing excipients.

More extensive experimental details regarding mucoadhesive nanocarriers presented in this section can be found in Table 1.

## 4. Beyond Bioadhesion: Mucus-Penetrating and Penetration-Enhancing Nanocarriers

### 4.1. Mucus-Penetrating Nanocarriers

**Figure 5:**
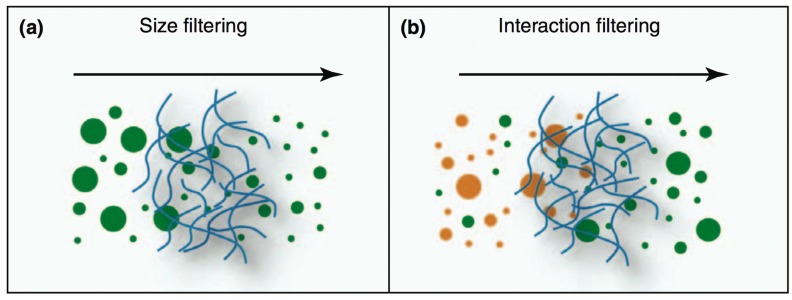
Major mechanisms hindering particles from diffusing through mucus: (**a**) Size filtering, by which only particles smaller than the mesh pores of the mucin fiber network can cross, whereas larger objects are blocked; (**b**) Interaction filtering, when particles behavior is different according to their surface properties: orange particles interacting strongly with the components of the mucus gel are trapped, whereas green particles showing weak interactions are allowed to go across (reproduced with permission from [121]).

The popular mucoadhesive approach has been recently challenged by the evidence of the multiple barrier properties provided by the mucus layer. The mucus acts as a dynamic semipermeable barrier via two major synergistic mechanisms: interaction filtering and size filtering (Figure 5) [120].

Interaction filtering occurs for molecules, supramolecular structures, and particles, independently of their size. This phenomenon limits diffusion through the mucus via direct non-specific interactions, such as electrostatic, hydrogen and hydrophobic binding with glycosylated and non-glycosylated regions of mucins as well as with lipid components of the mucus. These interactions were evidenced for charged and/or hydrophilic molecules, peptides and proteins, and lipophilic drugs [121].

Mucus is a dense molecular network with a characteristic mesh spacing that prevents larger particles’ diffusion through it. This mesh spacing has been reported by various authors to vary from 20 to 200–500 nm [120,122].

Hence, it has been hypothesized that the use of sufficiently small nanocarriers, coated with polymers like poly(ethylene) glycol (PEG) that minimize interactions with mucins, may increase mucus diffusivity and favor a close contact with the underlying epithelium. Interestingly, since 2004, the group of Maria José Alonso observed that when fluorescent labelled PEG–PLA nanoparticles were administered intranasally to rats, the presence of PEG, a small size (175 nm), and a high PEG coating density were in favor of particle accumulation within the nasal mucosa [123]. Later on, using multiple particle tracking, a group at John Hopkins University demonstrated that 100 and 200 nm particles coated with low *M*_W_ PEG (2 or 5 kDa) at high density, were able to rapidly penetrate the human respiratory [124] and chronic rhinosinusitis mucus [125], and designed mucus-penetrating PEG–PLGA nanoparticles [126,127]. However, those results were not replicated in subsequent experiments in which PEG-coated magnetic nanoparticles, despite their low adhesion to mucus components, failed to penetrate through native respiratory mucus even when applying a magnetic field [128].

In two different studies, diazepam and midazolam, two benzodiazepines used for the treatment of *status epilepticus*, were encapsulated in PLGA nanoparticles coated with poloxamers 407 (Pluronic^®^ F127), a block co-polymer surfactant previously reported to enhance the mucus penetration of nanoparticles [125]. In both cases, the optimal particles size was lower than 200 nm, they had negative surface potential (ζ potential between −15 and −30 mV), and displayed controlled drug permeation kinetics across sheep nasal mucosa, direct brain transport (DTP around 60%), and superior brain targeting efficiency compared to a drug solution after nasal administration to rats (235–260% vs. 125–160% DTE) [51,129]. The authors did not claim or study the mucus penetration of the nanoparticles, but the nanocarrier characteristics matched those of mucus-penetrating carriers.

Recently, Sekerdag and collaborators proposed lipid/PEG–PLGA nanoparticles as mucus-penetrating carriers of farnesylthiosalicylic acid (FTA) for a non-invasive nose-to-brain treatment of glioblastoma. Upon nasal administration, the nanoparticles significantly reduced the volume of a tumour induced by implanting RG2 cells in the rat brain (55% reduction by MRI analysis). The antitumor effect obtained was comparable to that obtained with the same nanoparticles administered intravenously. The biodistribution studies evidenced that the percentage of the drug dose reaching the brain was 0.04% of the injected dose normalized per tissue weight (ID/g) for both administration routes, but with nasal administration the dose fraction accumulating in liver and spleen was significantly reduced, suggesting a higher safety of the nasal treatment [130].

It is worth noticing that, in some cases, PEG-coated nanoparticles were used as mucoadhesive carriers [131], but this is because of the fine tuning of the particle size and coating properties required to obtain particles able to slip through the mucus layer [132]. Furthermore, PEG coating has been associated with reduced interactions with epithelial cells and consequent reduced uptake for the same reason of mucus penetration, i.e., reduced interactions with proteins and biomolecules [133,134]. It is clear that mucus-penetrating particles in nose-to-brain delivery require more robust studies to demonstrate the proof-of-concept in terms of improved CNS targeting compared to other approaches based on surface modification.

### 4.2. Penetration-Enhancing Nanocarriers

In alternative to mucus-penetrating “stealth” particles, some authors developed particles whose components can act as penetration enhancers. Actually, classifications often overlap (for example chitosan acts both as a mucoadhesive agent and as a penetration enhancer), but for the purpose of the present review, penetration-enhancing particles are considered those made of components, often surfactants, claimed to enable alterations of the barrier properties of the nasal mucosa.

Zolmitriptan and sumatriptan, two selective serotonin agonists in use for the treatment of acute migraine, were encapsulated into micelles composed of PEG 400, benzyl alcohol, Vitamin E TPGS, Pluronic^®^ F127, and Trascutol P^®^. In particular, Transcutol P^®^, i.e., diethylene glycol monoethyl ether, and Vitamin E TPGS were used to solubilize and enhance the absorption of the drugs through the nasal mucosa [135]. The micelles, whose size was lower than 25 nm, significantly increased triptan delivery to the brain (up to 3–7% of the administered dose) compared to IN or IV administration of a drug solution and did not show local toxicity, even after prolonged nasal administration in comparison to controls (28 days) [136,137].

Olanzapine-loaded nanocubic vesicles, obtained by incorporating the surface-active triblock copolymer poloxamer 188 (it is a hydrophobic polypropylene oxide block capped with hydrophilic polyethylene oxide moieties) in phosphatidylcholine bilayers, were compared with the corresponding liposomes in biodistribution studies in rats following nasal delivery. The nanocubic vesicles (size 363 nm, PDI 0.088, EE% 67) improved the absolute bioavailability (37.9% vs. 14.9%) and DTE (100% vs. 80%) compared with the control liposomal formulation. The improvement was attributed to the presence of poloxamers in the formulation, which conferred both higher elasticity and penetration-enhancing properties to the vesicles [138]. In this study, one of the few using LC–MS/MS to quantify the drug in the biodistribution studies, the nanocarrier nasal delivery was not superior to the IV control in delivering the drug to the brain, as evidenced by the value of the DTE. Similarly, in the work of Albdelrahman and co-workers, the elasticity of spanlastics nanovesicles was the claimed mechanism improving the N2B delivery of the antipsychotic drug risperidone. Spanlastics vesicles, produced by injecting an ethanol solution of Span 60 and risperidone into a PVA aqueous solution (size 103 nm, PDI 0.34, EE% 64), exhibited relatively high Newtonian viscosity, improved permeation through ex vivo nasal sheep mucosa, and improved brain accumulation of the drug in comparison to the non-encapsulated drug (DTE 469% versus 217%). However, the latter led to a higher DTP (55% vs. 79%), as spanlastics significantly improved the systemic absorption of the drug [139].

Gelatin-nanostructured lipid carriers were used for nose-to-brain delivery of a neurotrophic factor, i.e., basic fibroblast growth factor (bFGF), suggested to protect dopaminergic neurons in Parkinson’s disease. The gelatin nanoparticles were prepared by water-in-water emulsion in the presence of poloxamer 188 and phospholipids, crosslinking with glyceraldehyde, and subsequent freeze–drying. The nasal administration of the gelatin NLC significantly increased exogenous bFGF in the olfactory bulb and striatum without affecting the integrity of the nasal mucosa. The surface-modified nanocarriers outperformed control gelatin nanoparticles also in studies with hemiparkinsonian rats, inducing functional recovery after IN but not after IV administration. This was attributed to the presence of poloxamer 188 and its ability to reduce the barrier effect of the mucus layer by altering its viscosity and to enhance the permeation by interacting and perturbing lipid membranes and/or modulating tight junctions [140]. Polysorbate 80 (Tween 80) was claimed to have similar effects in SLN loaded with rosmarinic acid and made of glyceryl monostearate (GMS) and hydrogenated soy phosphatidylcholine. These particles were designed to manage the symptoms of Huntington’s disease. Polysorbate 80-coated SLN improved the behavioral abnormalities and levelled off the oxidative stress in rats treated with 3-nitropropionic acid to a greater extent compared with rosmarinic acid administered intranasally or to the same nanoparticles injected intravenously [141].

Fatty acids have been traditionally indicated as absorption-promoting agents in nasal delivery [142]. Recently, zolmitriptan was formulated in novasomes, i.e., nanovesicular fatty acid-enriched structures. The optimized novasomes formulated with a combination of Span^®^ 80, cholesterol, and stearic acid, showed enhanced brain accumulation (*C*_max_ 1.27% ID/g) with a direct transport percentage of 99.2% when compared with the intravenous drug solution. The effects were attributed to the potential disruption of the nasal membrane and to the ability of these vesicles to “squeeze” through the olfactory epithelium opening [143]. The actual contribution of this latter mechanism to the nose-to-brain delivery is far from being supported by substantial data demonstrating the claimed capability of elastic vesicles to transfer across the nasal mucosa. Similarly, the occlusive effect claimed by other authors for nasally delivered alprazolam SLN appears highly unlikely [144].

In general, the reviewed studies on penetration-enhancing nanocarriers lack the key control represented by the administration of a conventional formulation of the same drug containing the penetration enhancer alone. Only upon demonstration of a superior nose-to-brain delivery over this control, the use of nanocarriers could be fully warranted.

More extensive experimental details regarding mucus-penetrating and penetration-enhancing nanocarriers presented in this section can be found in Table 2.

## 5. Targeting the Nasal Epithelium for Optimizing Nose-to-Brain Delivery

Undoubtedly, one of the most fascinating aspects of pharmaceutical nanotechnology is the so called “active” targeting of nanoparticles, i.e., the recognition of ill cells or tissues by surface ligands able to interact specifically with receptors and/or other biomolecules present on the biological target. The specific delivery of the therapeutic drug dose to its site of action, avoiding side effects and optimizing efficacy, has been the Grail Quest of pharmacotherapy since the dawn of modern pharmaceutical sciences [145].

As pointed out in an excellent paper by Alexander Florence, several obstacles (often underestimated or disregarded by scientists) exist in vivo for targeted nanocarriers (nanocarrier aggregation, premature drug release from the carrier, uptake by the reticuloendothelial system, off-target delivery, degradation) and they have so far hindered the successful translation of the targeting approach in drug delivery to clinical settings [146].

As far as nose-to-brain delivery is concerned, interestingly, the approach is not directed to the delivery of the particles themselves to a specific cell or receptor within the CNS, but to their interaction with cells of the nasal region more likely to favor carrier translocation to the brain, i.e., the olfactory epithelium. Several surface ligands have been proposed, and the two most studied strategies have been lectins and cell-penetrating peptides for targeted nanocarriers.

### 5.1. Lectin-Modified Nanocarriers

Lectins are proteins or glycoproteins extracted from plants, able to recognize and bind with high specificity glycan arrays of glycosylated lipids and proteins present on the surface of different cell types. For this reason, since 1988, they have been proposed as targeting ligands for drug delivery systems, originally to obtain absorption enhancement in the gastrointestinal tract [147]. The potential of lectins for nose-to-brain delivery has been demonstrated qualitatively and quantitatively in two seminal works by Broadwell and Balin [148] and Thorne et al. [149]. Those works demonstrated that wheat germ agglutinin (WGA)–horseradish peroxidase conjugate (62 kDa) can bind the cell surface of olfactory sensory cells, undergo adsorptive endocytosis and anterograde axonal transport, and eventually accumulate in the olfactory bulb at concentrations (140 nM) more than 100-fold higher than those obtained by IV administration of the same conjugate and 700-fold greater than those attained by the enzyme not conjugated to WGA. In fact, WGA obtained from *Triticum vulgare* specifically binds to the abundant *N*-acetyl-d-glucosamine and sialic acid residues in nasal structures. Consequently, WGA has been the most explored targeting ligand for nanocarriers for nose-to-brain delivery.

WGA was conjugated to PEG–PLA nanoparticles to obtain 85–90 nm targeted nanoparticles loaded with the fluorescent dye 6-coumarin. WGA-targeted nanoparticles administered intranasally improved both blood (1.4-fold) and brain (twofold) concentrations of the fluorescent dye when compared to non-targeted PEG–PLA nanoparticles, without appreciable nasal ciliotoxicity [150]. Other studies in which WGA-conjugated PEG–PLA were radiolabeled with ^125^I revealed that, after nasal administration to rats, the nanoparticles were rapidly (5–30 min) transported to the CNS via the extracellular pathway along the olfactory and especially the trigeminal nerves, while the cerebrospinal fluid appeared to contribute less to the process [151].

**Figure 6:**
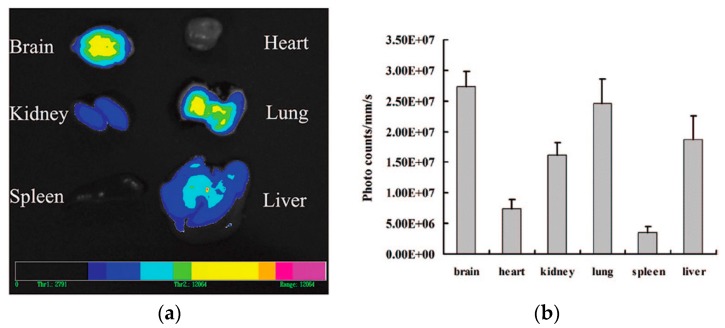
Distribution of wheat germ agglutinin (WGA)-conjugated quantum dots-loaded nanoparticles in various organs, 3 h after intranasal administration: (**a**) optical image (**b**) quantification of luminescence signals (adapted with permission from [153]. Copyright 2008 American Chemical Society).

The same group from the Fudan University of Shanghai (PR China) encapsulated the vasoactive intestinal peptide (VIP), a neuroprotective peptide potentially useful in several neurodegenerative disorders including AD, in WGA-conjugated PEG–PLA nanoparticles. In biodistribution studies upon nasal administration to mice, 30–50% of radioactively labelled VIP was found in the CNS, and WGA nanoparticle conjugation improved brain targeting (5.66–7.71-fold increase) compared to non-targeted PLA nanoparticles (3.57–4.74-fold increase) and control VIP solution. Furthermore, in an in vivo model of cholinergic impairment, VIP-loaded WGA-NP improved the spatial memory of rats at lower doses compared to non-targeted nanoparticles (12.5 versus 25 µg/kg) [152]. In a subsequent work, WGA-targeted PEG–PLA nanoparticles were loaded with quantum dots aiming to develop specific brain imaging agents for CNS diseases. Once again, the brain targeting capacity of the WGA was demonstrated with a relative fluorescence intensity detected three hours after nasal administration in mice (40 mg/kg, 5 µL each nostril) ranked as follows: brain ≥ lung > liver > kidney > heart > spleen (Figure 6) [153].

PEG–PLA or PEG–PLGA nanoparticles were conjugated to other lectins, such as *Solanum tuberosum* lectin (STL), *Ulex europeus* agglutinin I (UEA I), and odorranalectin (OL). The first binds to *N*-acetylglucosamine [154], and the remaining two bind to L-fucose, which is largely present in the olfactory epithelium [155,156]. STL-functionalized PEG–PLGA nanoparticles, loaded with haloperidol, were administered intranasally and increased the drug concentrations in the brain by 1.5–3-fold compared to non-STL-functionalized particles and other administration routes [157].

Since immunogenicity is one of the major problems of lectins, and odorranalectin (OL) is the smallest peptide with lectin-like activity, this molecule was identified as a potential targeting ligand with reduced immunogenicity. OL-conjugated PEG–PLG nanoparticles were loaded with urocortin, a peptide reported to restore nigrostriatal function in PD. In vivo studies in hemiparkinsonian rats evidenced an improvement of the symptoms of the dopaminergic lesion with OL-conjugated nanoparticles compared to control. This was shown to be related to a partial but incomplete recovery of monoamine neurotransmitter levels [158].

Nevertheless, lectins still face criticism related to their potential toxicity and immunogenicity. It is mandatory to perform a toxicological assessment of the lectin-targeted carrier both systemically and locally [159].

### 5.2. Cell-Penetrating Peptides as Surface Ligands for Targeted Nanocarriers

Cell-penetrating peptides (CPPs) are short cationic sequences of amino acids, able to cross cell membranes and translocate to the intracellular space according to the “model” represented by the HIV transactivator of transcription (Tat) protein [160]. CPPs have been shown to be non-specific to cell type and able to translocate various cargos (small molecules, proteins, nucleic acids, nanocarriers) across different biological barriers, such as intestinal wall, BBB, skin [161].

These characteristics make CPPs attractive as ligands for nose-to-brain delivery of nanocarriers. In the study by Gartziandia, the conjugation of CPPs (Tat or Penetratin) to the surface of polymer and lipid nanoparticles, improved their ability to cross an in vitro primary rat olfactory cell monolayer model [67]. In another study, PEG–PLA nanoparticles functionalized with low-molecular-weight protamine, were found to accumulate in 16HBE14o- human bronchial epithelial cells to a greater extent than the unmodified particles. After loading with the fluorescence dye coumarin-6 and nasal administration to rats, they increased fluorescence in various brain structures more than twofold compared to the unmodified particles [162]. In a similar experiment, micelles obtained using Tat conjugated to methoxypoly(ethylene glycol)-poly(**ε**-caprolactone) (mPEG–PCL) amphiphilic block copolymers and loaded with coumarin, accumulated more than original micelles in C6 rat glioma cells and in rats bearing intracranial C6 tumors. In the latter in vivo experiment, no difference between micelles was found in the fluorescence brain distribution 1h after nasal administration. However, after 4 h, the fluorescence signal decreased for the unmodified micelles, whereas it increased in animals treated with Tat-conjugated mPEG–PCL micelles. This was probably due to their greater ability to penetrate intracellularly [163]. In subsequent works, the same group from Tokyo University of Pharmacy and Life Sciences loaded the abovementioned micelles with camptothecin to treat brain tumors and with small-interfering RNA (siRNA) for the suppression of genes involved in CNS disorders. Camptothecin-loaded Tat-mPEG–PCL micelles showed a higher cytotoxic effect in rat C6 glioma cells in vitro compared to micelles not modified with the CPP. More interestingly, after nasal administration, they prolonged the survival of rats bearing intracranial C6 glioma, compared to the control micelles and a drug solution (1.2 mg/kg, once-a-day for one week) [164]. In the case of siRNA, the nucleic acid was condensed with Tat-mPEG–PCL to obtain polyplexes of 50–100 nm. Brain distribution studies after nasal administration, carried out after loading the micelles with Alexa-dextran 10 kDa as a model of siRNA, evidenced an increase in brain accumulation in comparison to the controls (IV Tat-mPEG–PCL micelles or IN mPEG–PCL micelles). This resulted from the Tat-promoted micelle permeation of the nasal mucosa and increased uptake within the olfactory and trigeminal nerves [165].

Despite the translocation abilities, CPPs have some drawbacks, such as sequestration in endosomes, limited intracellular localization, and lack of efficient transport to the cytoplasm [160]. These drawbacks have prompted the development of different delivery approaches.

For example, the group of Paolo Giunchedi from the University of Sassari designed a drug delivery system for N2B delivery of the complex between a 29-amino-acid peptide, derived from the rabiesvirus glycoprotein (RVG), and siRNA interfering with the expression of BACE1. This β secretase is responsible for the processing of the amyloid precursor protein in the β amyloid peptide, the main component of extracellular plaques that are a hallmark of AD. The RVG–siRNA complex was loaded into SLN coated with chitosan; preliminary experiments showed that RVG–siRNA encapsulated in SLN, particularly if coated, crossed a Caco-2 cell monolayer more efficiently than the naked complex. Thus, the mucoadhesive nanocarrier protects the RVG–siRNA complex and helps it cross the mucosal barrier enabling the interaction of the RVG cell-penetrating peptide with the acetylcholine receptors located in the trigeminal nerve ending and olfactory bulb. This approach has yet to be validated by in vivo experiments [166].

### 5.3. Other Targeting Approaches

Several other ligands may be potentially useful to enhance nose-to-brain delivery. This section provides some examples of the most interesting alternatives to lectins and cell-penetrating peptides.

One of the early approaches for targeted drug delivery, also suggested for other administration routes, has used viral vectors. Frenken and Solomon chose filamentous bacteriophages as vectors for the nasal administration of anti-β amyloid (Aβ) antibodies, designed for monitoring amyloid plaques in living AD patients. The bacteriophage f88 was genetically engineered to encode protein III on its surface. This protein is a single-chain antibody derived from variable sections of the light and heavy chains of the anti-Aβ IgM 508 antibody. After three daily nasal administrations of the filamentous bacteriophage vector, amyloid plaques were successfully targeted and visualized using fluorescent-labeled antiphage antibodies in the olfactory bulb and the hippocampus region of transgenic mice carrying a double mutation in the amyloid precursor protein (APP). The passage across the nasal barrier was attributed to the linear structure of the phage, whose penetration properties had been already demonstrated. The lack of spreading to other brain sections suggested a transport via the olfactory neurons. The vector was proven inert and non-toxic, nevertheless the trigger of immune defense mechanisms, such as the activation of microglia scavenger cells, could be one of the possible drawbacks [167]. Interestingly, the approach is the object of a filed patent application [168].

Lactoferrin (Lf), a natural iron-binding cationic glycoprotein of the transferrin family, has been used as targeting ligand of nanocarriers, as Lf receptors are highly expressed on the surface of respiratory epithelial cells as well as on neurons and brain endothelial cells [169,170]. For this reason, lactoferrin-modified PEG–PCL nanoparticles were developed to enable brain delivery following intranasal administration of the neuroprotective NAP peptide, a fragment of the activity-dependent neuroprotective protein. Nanoparticles were prepared using the emulsion/solvent evaporation technique followed by conjugation with thiolated Lf. The Lf-targeted nanoparticles increased brain accumulation more than twofold compared to the unmodified nanoparticles. In addition, they showed improved neuroprotective effects in an AD model, i.e., mice intracerebroventricularly co-injected with ibotenic acid and β-amyloid_1–40_, as shown by behavioral experiments such as the Morris water maze task. This was related to an amelioration of the impaired cholinergic neurotransmission via a reduction of acetylcholinesterase activity and reduced depletion of choline acetyltransferase [171]. The same targeting strategy was successfully adopted for rotigotine-loaded PEG–PLGA nanoparticles for PD treatment [172] and for mPEG–PLA nanoparticles encapsulating α-asarone. This compound is extracted from the traditional Chinese medicine herb *Acorus tatarinowii* Schott, recently proposed for the treatment of epilepsy [173].

PEG–PLA nanoparticles loaded with the analgesic peptide α-cobrotoxin were modified with OX26 antibodies to target transferrin receptors in the BBB. The results showed that brain delivery of the peptide labelled with fluorescein isothiocyanate was enhanced by the intranasal delivery of nanoparticles in comparison with intramuscular administration and that the enhancement was more pronounced in the case of antibody-targeted nanoparticles. The peptide solution could barely penetrate the brain. Despite the authors attributed this result to the capacity of the nanoparticles to cross the BBB, the hypothesized mechanism of transport, i.e., intact nanoparticles crossing the nasal respiratory mucosa, entering capillaries, and finally crossing the BBB, was not supported by any experimental evidence [174].

Finally, in an interesting approach from the group of Rodney Ho of the University of Washington (USA), liposomes targeted with the integrin-targeting ligand Arg–Gly–Asp (RGD) were coupled with a pressurized olfactory drug (POD) delivery device to improve nose-to-brain administration of the analgesic opioid fentanyl. RGD can increase binding and enhance permeability across epithelial cells expressing α_v_β_3_ integrins. Thus, fentanyl-loaded liposomes integrating the palmitoylated peptide, were used to prolong the residence time and enhance the absorption of the nasally administered opioid. The POD intranasal delivery device is a new device enabling the preferential deposition of the aerosolized formulation in the olfactory region. Experiments demonstrated that RGD-conjugated liposomes could withstand the aerosolization with the POD device without size change, phospholipid bilayer disruption, or impairment of the targeting properties. Interestingly, when the liposomes were administered to rats using the POD device, fentanyl plasma concentrations as well as those measured in the brain 5 min post-administration were lower than those obtained using free fentanyl (although not significantly different). However, when the analgesic effect was measured, the fentanyl-loaded RGD liposomes provided a slightly slower onset of action and a lower, but more prolonged, analgesic effect [175]. The decision to use in small animals a device optimized for deposition in the human nasal cavity is questionable. A larger animal model such as sheep would be more appropriate.

More details about targeted nanocarriers for nose-to-brain delivery are presented in Table 3.

## 6. Future Perspectives of Nose-to-Brain Delivery with Nanocarriers

Nose-to-brain delivery is a fascinating scientific topic. In the quest to achieve a non-invasive, efficient, safe, and potentially disruptive innovation in the treatment of CNS disorders and brain diseases, the application of nanocarriers appears as an asset, with several advantages but also few risks to be addressed early-on in the medicinal product development. Despite the promising results with several drugs, different materials and targeting approaches, to the best of our knowledge not a single one is being actively developed by a pharmaceutical company and no technology has been transferred from the laboratory to the clinical stage. Various reasons lay behind the lack of translational research successes for nanomedicines, among which manufacturing scale-up and safety and quality challenges related to these non-biological complex medicinal products. Other reasons have to be pinpointed in some shortcomings of the scientific studies that in several cases highlighted the potential of the approach and overlooked the weak spots or missed the proper controls to demonstrate a real superiority over “conventional” formulations. Furthermore, often the drugs selected are already able to cross the BBB to some extent and/or produce CNS pharmacological effects. In such cases, nanoencapsulation can only improve the performance. Nanomedicines, especially in the case of a critical application such as the therapy of CNS conditions, are not required to *improve*, but to *enable* therapies that would not be possible without the application of nanoencapsulation [111].

For these reasons, the planning of future nose-to-brain research protocols in drug nanocarriers should take into account the following points in order to demonstrate the superiority of the designed nanomedicines and provide the data necessary for further development of a medicinal product:

- Select a potent drug with unfavorable physicochemical characteristics for N2B;
- Design particles with biocompatible, biodegradable, Generally Recognized as Safe (GRAS) materials;
- Adopt a robust, validated, and up-scalable fabrication method;
- Determine drug release from nanocarriers in biorelevant conditions;
- Establish early on the safety and biodegradability pattern of the nanocarrier;
- If possible/advisable, adopt particles with sizes of 100–400 nm, as smaller ones are more likely to enter the CNS with consequent concerns related to the nanotoxicology of those materials;
- Develop bioanalytical methods able to detect the drug, instead of fluorescent or radioactive labels, in biodistribution studies, if possible;
- Develop methods allowing to track the particles in the tissues in order to differentiate free drug and nanomaterial biodistribution;
- Carry out the in vivo experiments perfusing the organs before dissection, in order to eliminate blood contamination from the analytical quantitation;
- Establish the pharmacokinetics of the free and nanoencapsulated drug, applying multiple and relevant controls (IV and IN administered solutions or formulations including absorption-promoting excipients);
- Determine relevant parameters such as drug targeting efficiency (DTE) and direct transport percentage (DTP);
- Establish the therapeutic proof of concept through pharmacodynamics studies in a disease model as close as possible to the human condition;
- Combine PK and PD data to critically predict the feasibility of the treatment in terms of drug dose, amount of formulation to administer, posology, etc.;
- Select the candidate formulation for pre-clinical/clinical development.

In conclusion, nose-to-brain delivery evolved from a series of interesting observations looked upon with skepticism to a promising, although challenging, field of research. Nanomedicines appear to be a pivotal tool to enable the brain delivery of potent drugs unable to cross the BBB and, when used as such, they will fulfill the potential demonstrated in the many scientific studies conducted so far.

## Figures and Tables

**Table 1:** Mucoadhesive nanocarriers studied for nose-to-brain delivery.

Nanocarrier	Drug	Application	Size (nm)	PDI	ζ-potential (mV)	EE (%)	DL (%)	Biodistribution	DTE	DTP	Ref.
Methacrylic-PCL Ncps	Olanzapine	Schizophrenia	254.9 ± 12.1	0.030	+22.0 ± 1.2	99.0	-	HPLC-UV	-	-	[88]
β CD Albumin NPs	Tacrine	AD	189.3 ± 10.0	0.228	−10.2 ± 0.6	-	12.5	-	-	-	[90]
*Delonix regia* gum NLC	Ondansetron	Nausea	92.3 ± 13.0	0.360	−11.5 ± 2.3	39.5	5.6	HPLC-UV	506.0	97.1	[91]
Alginate NPs	Venlafaxine	Depression	173.7 ± 2.5	0.391	+37.4 ± 1.7	81.3	26.7	CFM	425.8	76.5	[92]
Chitosan NPs	Estradiol	AD	269.3 ± 31.6	-	+25.4± 0.7	64.7	1.9	HPLC-Fluo	320.0	68.4	[95]
Chitosan NPs	Rivastigmine	AD	185.4 ± 8.4	0.391	+38.4 ± 2.8	85.3	43.4	CFM	355.0	71.8	[96]
Chitosan NPs	Thymoquinone	AD	172.4 ± 7.4	0.130	+30.3 ± 2.2	63.3	31.2	γ scintigraphy	3321.2	97.0	[97]
Chitosan NPs	Bromocriptine	PD	161.3 ± 4.7	0.440	+40.3 ± 2.7	84.2	37.8	γ scintigraphy	633.0	84.2	[98]
Chitosan NPs	Ropinirole	PD	173.7 ± 2.3	0.390	+32.7 ± 1.5	69.6	13.8	γ scintigraphy	-	-	[99]
Chitosan NPs	Rasagiline	PD	151.1 ± 10.3	0.380	-	96.4	-	HPLC-UV	325.0	69.3	[100]
Chitosan NPs	Pramipexole	PD	292.5 ± 8.8	0.292	+14.0 ± 2.9	93.3	-	-	-	-	[101]
Chitosan NPs	Tapentadol	Chronic pain	201.2 ± 1.5	0.201	+49.3 ± 1.2	63.5	17.2	HPLC-UV	321.0	68.8	[102]
Trimethylchitosan NPs	Leu-Enk	Chronic pain	443.0 ± 23.0	0.317	+15.0 ± 2.0	78.3	14.0	-	-	-	[103]
Thiolated Chitosan NPs	Tizanidine	Muscular pain	276.2 ± 13.9	-	+18.3 ± 1.4	75.6	-	γ scintigraphy	8523	98.8	[105]
Thiolated Chitosan NPs	Cyclobenzaprine	Muscular pain	272.1 ± 11.5	-	+20.9 ± 1.7	70.4	5.4	γ scintigraphy	2471	96.0	[106]
Thiolated Chitosan NPs	Selegiline	Depression	215.0 ± 34.7	0.214	+17.1	70.0	-	-	-	-	[107]
GC SBE β CD NPs	Dopamine	PD	372.0 ± 81.0	0.260	+9.3 ± 1.3	54.5	-	FM	-	-	[108]
Chitosan-PLGA NPs	Chlorpromazine	Schizophrenia	463.9 ± 12.0	0.187	+21 ± 2	36.7	4.6	-	-	-	[109]
Chitosan-coated Liposomes	Ghrelin	Cachexia	194.0 ± 6.1	0.198	+6.0 ± 0.4	56.1	-	-	-	-	[110]
Lecithin/Chitosan NPs	Simvastatin	AD	204.5 ± 15.4	0.098	+48.4 ± 4.1	98.5	-	γ scintigraphy	-	-	[113]

Abbreviations: AD, Alzheimer’s Disease; CFM, Confocal Fluorescence Microscopy; EE, Encapsulation Efficiency; GC SBE β CD NPs, Glycol Chitosan Sulfobutylether-β-cyclodextrin Nanoparticles; Leu-Enk, Leucine-Enkephalin; FM, Fluorescence Microscopy; NCL, Nanostructured Lipid Carriers; PCL Ncps, Poly(ε-caprolactone) Nanocapsules; PD, Parkinson’s Disease.

**Table 2:** Mucus-penetrating and penetration-enhancing nanocarriers studied for nose-to-brain delivery.

Nanocarrier	Drug	Application	Size (nm)	PDI	ζ-potential (mV)	EE (%)	DL (%)	Biodistribution	DTE	DTP	Ref.
Pluronic^®^ F127 PLGA NPs	Diazepam	Epilepsy	183.2	<0.200	<−15	87.8	-	γ scintigraphy	258.0	61.0	[51]
Pluronic^®^ F127 PLGA NPs	Midazolam	Epilepsy	164.0 ± 4.5	0.099	−16.6 ± 2.5	87.4	5.3	γ scintigraphy	234.7	-	[129]
Lipid/PEG-PLGA NPs	FTA	Glioblastoma	164.3 ± 10.3	0.192	−12.0 ± 1.3	97.7	3.5	HPLC-MS	-	-	[130]
TPGS Micelles	Zolmitriptan	Migraine	24.2 ± 0.7	0.064	-	-	-	γ scintigraphy	-	-	[136]
TPGS Micelles	Sumatriptan	Migraine	23.1 ± 0.4	0.046	-	-	-	γ scintigraphy	-	-	[137]
Poloxamer 188 Cubosomes	Olanzapine	Schizophrenia	363.0 ± 31.8	0.088	-	67.3	-	HPLC-MS/MS	100	-	[138]
Spanlastics	Risperidone	Schizophrenia	103.4	0.341	−45.92	63.9	-	HPLC-MS/MS	468.9	55.2	[139]
Gelatin NLC	bFGF	PD	172.0 ± 1.3	0.105	−27.6 ± 1.1	86.7	4.6	Western blot	-	-	[140]
Polysorbate 80 SLN	Rosmarinic acid	HD	149.2 ± 18.2	0.290	−38.27	61.9	-	HPLC-UV	-	-	[141]
Novasomes	Zolmitriptan	Migraine	149.9 ± 10.9	0.477	−55.6 ± 1.0	92.9	-	γ scintigraphy	-	99.2	[143]

Abbreviations: bFGF, basic Fibroblast Growth Factor; EE, Encapsulation Efficiency; FTA, farnesylthiosalicylic acid; HD, Huntington’s Disease; NLC, Nanostructured Lipid Carriers; PD, Parkinson’s Disease; SLN, Solid Lipid Nanoparticles.

**Table 3:** Targeted nanocarriers studied for nose-to-brain delivery.

Nanocarrier	Drug	Application	Size (nm)	PDI	ζ-potential (mV)	EE (%)	DL (%)	Biodistribution	DTE	DTP	Ref.
WGA PEG-PLA NPs	VIP	AD	100–120	-	-	70.1	1.4	Radiolabeling (^125^I)	-	-	[152]
WGA PEG-PLA NPs	Quantum Dots	Brain Imaging	95.3 ± 41.0	-	−22.7 ± 1.2	-	-	Luminescence	-	-	[153]
STL PEG-PLGA NPs	Haloperidol	Schizophrenia	132 ± 20	0.174	−14.4 ± 0.1	73.2	0.85	HPLC	-	-	[157]
OL PEG-PLGA NPs	Urocortin	PD	114.8 ± 5.6	0.193	-	75.5	0.14	Fluorescence imaging	-	-	[158]
Tat mPEG-PCL Micelles	Camptothecin	Glioma	88.5 ± 20.2	-	10.4 ± 2.8	62.5	-	-	-	-	[164]
Tat mPEG-PCL Micelles	siRNA	CNS Disorders	51.0	-	11.3	-	-	Fluorescence imaging	-	-	[165]
RVG SLN Chitosan	siRNA	AD	358.4 ± 25.9	0.028	+10.5 ± 0.8	75.5	0.14	-	-	-	[166]
Lactoferrin PEG-PCL NPs	NAP	AD	88.4 ± 7.8	0.220	−23.6 ± 1.0	47.61	0.62	Fluorescence imaging	-	-	[171]
Lactoferrin PEG-PCL NPs	Rotigotine	PD	122.0 ± 19.3	0.194	−21.3 ± 2.2	92.6	~7	Fluorescence imaging	-	-	[173]
Lactoferrin PEG-PCL NPs	α-Asarone	Epilepsy	360.1 ± 3.7	0.165	−21.8 ± 1.0	86.3	7.3	UPLC-MS	261–734	>80	[173]
mAb OX26 PEG-PLA NPs	α-Cobrotoxin	Pain	96.2 ± 6.3	0.112	−33.4 ± 1.2	82.1	-	Fluorescence analysis	-	-	[174]
RGD Liposomes	Fentanyl	Pain	96.5 ± 6.1	-	-	~80	1.4	HPLC-MS	-	-	[175]

Abbreviations: AD, Alzheimer’s Disease; EE, Encapsulation Efficiency; NAP, NAPVSIPQ peptide; OL, Odorranalectin; STL, *Solanum tuberosum* Lectin; PD, Parkinson’s Disease; RVG, Rabiesvirus Glycoprotein; VIP, Vasoactive Intestinal Peptide.
